# Clarifying the role of clinical research nurses working in Sweden, using the Clinical Trial Nursing Questionnaire – Swedish version

**DOI:** 10.1002/nop2.1260

**Published:** 2022-06-02

**Authors:** Beatrice Backman Lönn, Senada Hajdarevic, Niclas Olofsson, Åsa Hörnsten, Johan Styrke

**Affiliations:** ^1^ Department of Nursing Umeå University Umeå Sweden; ^2^ Department of Research & Development Region Västernorrland, Sundsvall Hospital Sundsvall Sweden; ^3^ Department of Health Science Mid Sweden University Östersund Sweden; ^4^ Department of Surgical and Perioperative Sciences, Urology and Andrology Umeå University Umeå Sweden

**Keywords:** clinical research nurse, clinical study coordinator, clinical trial nurse, clinical trial nursing questionnaire, competence, nurse, professional development, registered nurse, role, swedish, tasks

## Abstract

**Aim:**

To explore the role of CRNs in Sweden and differences in competences and tasks, using the Clinical Trial Nursing Questionnaire – Swedish version (CTNQ‐SWE).

**Design:**

A cross‐sectional survey.

**Methods:**

Participants were identified through strategic sampling. Data were analysed by descriptive and comparative statistics.

**Results:**

The respondents were experienced nurses who felt proficient in their role, they felt more acceptance by the principal investigators than by nursing colleagues. A majority of CRNs are involved in all procedures specified in the CTNQ‐SWE. The most often performed tasks, also rated as the most important by the CRNs, concerned informed consent and management of investigational products. The education was often informal: with a lack of job descriptions and professional development plans. Need of formal specialist education was expressed.

**Conclusions:**

Knowledge about the role description can be used by clinical research enterprise internationally and healthcare organizations aiming to support CRNs in their role.

## 1 INTRODUCTION

A registered nurse (RN) can be involved in clinical research by working as a clinical research nurse (CRN). Globally, they are important in clinical research along with various experts from the multidiscipline research team. However, new competence and skills are required for the RNs who become CRNs, whose professional role is positioned between the care of patients and the requirements of the study protocol (Bevans et al., 2011; McCabe et al., 2019). International reports on the working tasks of CRNs are diverse, ranging from conducting simple study‐related tasks to being involved in study planning and development of study protocols, data collection, coordination and evaluation of studies, as well as presenting results at conferences and writing articles together with the researchers (Bevans et al., 2011; Brinkman‐Denney, 2013; Fowler & Stack, 2007; Purdom et al., 2017).

Even if their role is essential in clinical research, there exists no standardized, internationally agreed definition on the CRN role (Hastings et al., 2012). RNs struggle with their identity when becoming CRNs, trying to balance demands related to their responsibility for patient care and simultaneously following the study protocol. Adapting to the new role may create feelings of ambiguity and of being a novice again: to feel proficient takes time (Tinkler et al., 2018).

## 2 BACKGROUND

CRNs not only manage many complex situations in their daily work but also see to several different tasks and demands associated with the role of being a clinical working RN versus a CRN. Various challenges associated with the transition to the new role as CRN include the use of new terminology and involvement in complex studies, which may create feelings of being a novice again and cause discomfort and moral distress (Höglund et al., 2010; Tinkler et al., 2018).

Developing and using standards for CRNs is something that may improve the quality of clinical research and may require preparedness among RNs for the transition to their new role as a CRN. CRNs in the United States have since 2015 been recognized as a nursing specialty approved by the International Association of Clinical Research Nurses (IACRN), the American Nurses Association (ANA) and the National Institutes of Health (https://www.iacrn.org, 2018). No such standards or approvals exist in Sweden, but in 2013 the Swedish government established a national committee and regional nodes for national coordination of clinical studies with a mission to support all research actors, including CRNs, with study support, education and consultancy (SOU 2013: 87). The purpose of the national coordination was to strengthen cooperation between health care, academia and industry in clinical studies. Since then, the regional nodes have given more attention to CRNs' role through both education and support from networks to improve the quality of clinical research. The lack of clear definitions and standards concerning professional competence and roles for CRNs is still a challenge, and there is a need for national consensus concerning the CRNs role, tasks and need of education.

The theoretical framework of this study is transition. According to Meleis et al. (2000), the mastery of the knowledge to perform a role is known as role clarification. A lack of role clarification can have a negative effect on role performance and relates to transitions, in this case from a RN to a CRN. The transitions between different professional roles and contexts are often complex and multidimensional and develop through phases (Meleis et al., 2000). How well the transition process proceeds, depends on personal, environmental and interpersonal resources (Young & Wilkersen, 2000).

In summary, there is a lack of studies of the Swedish CRN's role. A better understanding of the role, tasks, competence and skills could potentially create better conditions for the transition to a CRN. Since there is a lack of consensus concerning the CRN's role, we need to investigate the tasks they are performing and if there are differences in frequency and their views of the importance of these work tasks. This knowledge is also important for international comparison as well as for the quality of clinical research and to inform the national, regional research nodes and healthcare organizations, which are expected to support and educate the CRNs.

The aim of this study was therefore to explore the role of CRNs in Sweden and the differences in competences and tasks, using the validated Clinical Trial Nursing Questionnaire – Swedish version (CTNQ‐SWE).

## 3 METHODS

### 3.1 Design

A cross‐sectional study was conducted and data were collected using the Swedish version of the CTNQ‐SWE (Backman Lönn et al., 2019). This article adheres to the EQUATOR guidelines of reporting research using the STROBE checklist for observational research (Appendix S1), (Von Elm et al., 2008).

#### 3.1.1 Instrument

The CTNQ‐SWE is a validated questionnaire based on the Clinical Trial Nursing Questionnaire (Ehrenberger & Lillington, 2004) and has previously been translated into Swedish as well as adapted to Swedish organizational settings and demographics. The Swedish version was tested for face and content validity as well as reliability (reproducibility) with test–retest procedures. Face and content validity was achieved by the bilingual expert and the expert panel, furthermore reliability analysis was made for comparing responses in frequency and importance scales. It resulted in Cronbach's alpha coefficient for frequency scale 0.97 and for importance scale 0.97. Test–retest reliability/reproducibility, was made by a sample of *n* = 49 clinical research nurses in Sweden, responded on the web‐survey questionnaire on two different occasions. For the frequency scale, a correlation coefficient of .89 was obtained, and for the importance scale of 0.88 was obtained (Backman Lönn et al., 2019). The CTNQ‐SWE contains 159 items in 10 sections (See Table 1). Sections 1–8, with 120 items, examine the role components of clinical research that the CRNs are involved in.

**TABLE 1:** The content of each section in the CTNQ‐SWE

Sections	Items/ section (no)	Content
1. Protocol assessment	16	Assessment of protocols under development before study start, including budget and other resources such as settings and equipment; assessing the protocol for clarity and potential risk for study patients
2. Protocol planning	14	Preparing specific study documents such as case report forms and checklists; educating and informing study staff about Swedish regulations and laws; attending study meetings
3. Subject recruitment	15	Applying strategies in the recruitment procedure, such as development of recruiting materials (advertising, information materials, etc.); prescreening and screening of potential study participants
4. Informed consent process	14	Ensuring, for example, the correct use of language in the study information, that the patient has understood and correctly signed the consent, that a copy has been given and that consent was obtained before study participation
5. Investigational product	10	Management of study product/study drugs such as handling, documenting and administering the investigational product
6. Implementation and evaluation	23	Implementing activities according to study protocols, which includes routines, processes and implementing nursing actions for the study participants. Evaluation applies to follow‐up, nursing measures, side effects and adverse events (AE, SAE) as well as related communication with the study team
7. Data management	18	In all forms, managing source data, establishing records for study data, documenting deviations and participating in audit/inspections or reviewing and preparing documents for this
8. Professional nursing role performance	10	Activities CRNs perform in the role based on the professional perspective, including skills development, both on their own and how to contribute to others
9. Professional nursing role perception	10	Perception of the role
10. Sociodemographics	29	Years of practice, involvement in various kinds of studies and level of education

Different areas of activities and responsibilities for the CRNs are rated for *frequency* (how often they perform the task/area) and for *importance* (how important they perceive the task/area for the practice of CRNs). A five‐grade scale is used for: (a) the *frequency* of performing the activity (0, never, not part of my role; 1, once or twice; 2, occasionally when needed; 3, repeatedly, at various times; 4, extremely frequently) and (b) for the *importance* of the activity (0, not important; 1, somewhat important; 2, important; 3, moderately important; 4, very important). Section 9 contains 10 items concerning the professional nursing role perceptions. These answers are scored on a Likert scale, ranging from 1 (strongly agree) to 4 (strongly disagree) or the alternative response “not applicable to the role.”

While the original CTNQ includes three further sections: 10–12 with 24 items concerning the professional nursing role characteristics, organizational characteristics and demographic characteristics, the Swedish version has only one contextual section with 29 items concerning demographics, academic degree, certification level of experience in nursing and as CRN, employment status and work settings.

### 3.2 Data collection

**FIGURE 1:**
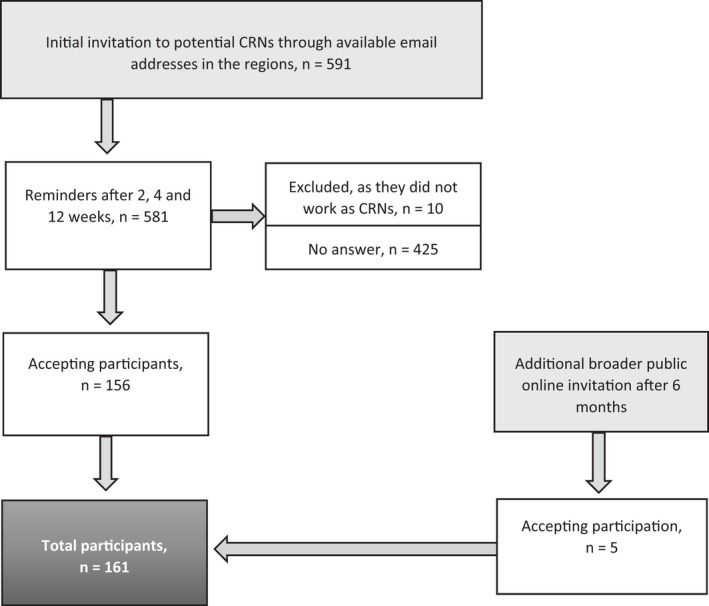
Flow chart of the sampling procedure

Since no Swedish register of a number of RNs working as CRNs exists, we did not know how many CRNs in total were eligible. Therefore, a request for email addresses of CRNs along with an invitation for participation was sent to all regions/county councils; LIF (Läkemedelsindustriföreningen, a trade association for the research‐based pharmaceutical industry); municipalities responsible for health care and research; national and regional nodes for clinical research; and lastly, the trade union for nurses. The study information was also shared through networks on LinkedIn. This resulted in a total of 591 email addresses representing potential participants. Eligible Swedish‐speaking RNs who worked or had worked as CRNs, study coordinators or clinical trial nurses were included. Professionals with educational backgrounds other than nurses, and who worked as clinical research assistants, were excluded. The strategic sampling process striving to reach all eligible CRNs in Sweden, is described in Figure 1.

The total study population (*n* = 591 email addresses) was invited to fill in the CTNQ‐SWE through a personal email link. The study information highlighted that the study concerned only those who were RNs working as CRNs. Ten persons who responded that they did not work as a CRNs were excluded. Reminders were sent out on three occasions, after 14 days, 1 and 3 months. Of the 581 potential participants, 156 people answered the questionnaire. Additionally, after 6 months, as an attempt to reach out to more participants, a public online link invitation was sent out to the regional nodes for distribution in their networks, that is, strategic sampling in two steps. Information about how many people in total were reached by the regional nodes in this step is lacking, but it resulted in only five additional responses from new participants. In total, 161 participants (28%) finally answered and sent back the CTNQ‐SWE (see Figure 1). The participant information is presented in Table 2.

**TABLE 2:** Characteristics of the study sample of CRNs in Sweden

Participants and characteristics *N* = 161	
Work experience. Mean (SD)	
Registered nurse, years	22.1 (10.2)
Clinical research nurse, years	9.87 (7.54)
Gender, *n* (%)	
Women	153 (95)
Men	8 (5)
Demographic area of Sweden; *n* (%)	
Northern part	25 (16)
Middle part	81 (50)
Southern part	55 (34)
Nursing academic degree; *n* (%)	
Diploma (older education)	22 (14)
Bachelor's degree	78 (48)
Master's degree (1 year)	30 (19)
Master's degree (2 years)	4 (2.5)
PhD	4 (2.5)
Nurse specialist education	19 (12)
Type of practice as CRN; *n* (%)	
Primary care	66 (41)
Hospital	91 (57)
University	4 (2)
Working time per month as CRN; *n* (%)	
Full‐time	76 (47)
Part‐time	85 (53)
Types of studies; *n* (%)	
Industry‐sponsored studies	126 (78)
Treatment studies	114 (71)
Quality of life studies	48 (30)

### 3.3 Data analysis

Data were analysed using SPSS Statistics version 23 and SPSS version 25 (SPSS Inc., Chicago, IL, USA). All items were analysed descriptively, and mean values and standard deviations are presented for sections 1 to 9 in Tables 3 and 4.

**TABLE 3:** Rated frequency (0–4) of activities among Swedish CRNs (*n* = 161) based on CTNQ‐SWE

Section 1–8	Mean per section	*SD*	Highest vs. lowest scored item (mean)
Protocol assessment	1.97	0.92	Identify concerns about the study (2.88) vs. Assess the study budget and economics (0.98)
Protocol planning	2.06	0.96	Participate in study initiation meetings (3.32) vs. Prepare and send documents to the ethical review authority (0.58)
Subject recruitment	2.61	0.76	Maintain logs for screening and monitoring for correct inclusion (3.66) vs. Develop study‐related recruitment materials such as print materials and website (1.10)
Informed consent process	3.24	0.59	Verify that written informed consent was obtained before each subject's participation in the study (3.85) vs. Assess the potential subject's goals and purposes for participation in a study (2.06)
Investigational product	3.19	1.08	Maintain accountability for the investigational product at the study site (3.39) vs. Teach study staff about the management of study product/study drugs (2.42)
Implementation and evaluation	2.87	0.71	Schedule and perform clinical procedures and tests according to protocol requirements (3.68) vs. Perform psychosocial assessments of the subject/family (1.7)
Data management	2.04	0.66	Enter data into case report form and confirm its accuracy (3.76) vs. Prepare final written reports for the ethical review authority or sponsors upon study completion (0.20)
Professional nurse role performance	2.02	0.75	Seek additional experiences to maintain and expand knowledge of clinical research and nursing expertise (2.99) vs. Identifying clinical problems that may be appropriate to research (1.37)

Abbreviation: SD, Standard deviation.

**TABLE 4:** Rated importance (0–4) of activities among Swedish CRNs (*n* = 161) based on CTNQ‐SWE

Section 1–8	Mean per section	(SD)	Highest vs. lowest scored item (mean)
Protocol assessment	2.49	0.87	Consider the ability to maintain study patients' security and well‐being (3.31) vs. Assess the study budget and economics (1.50)
Protocol planning	2.53	0.98	Participate in study meetings (3.68) vs. Prepare and send documents to the ethical review authority (0.58)
Subject recruitment	2.84	0.75	Maintain subject enrolment logs (3.68) vs. Develop study‐related documents (1.57)
Informed consent process	3.42	0.56	Verify that written informed consent was obtained before participation (3.89) vs. Assess the potential subject's goals and purposes for participation (2.22)
Investigational product	3.51	0.82	Maintain accountability and ensure that the investigational product is used only in accordance with the approved protocol (3.66) vs. Teach staff about management of study product (3.03)
Implementation and evaluation	3.22	0.66	Schedule and perform clinical procedures and tests according to protocol requirements (3.83) vs. Perform psychosocial assessments of the subject/family (2.20)
Data management	2.50	0.80	Enter data into case report form and confirm its accuracy (3,74) vs. Prepare final written reports for the ethical review authority or sponsor upon study completion (0.61)
Professional nurse role performance	2.73	0.84	Seek additional experiences to maintain and expand knowledge of clinical research and nursing expertise (3.52) vs. Identify clinical problems that may be appropriate to research (2.08)

Abbreviation: SD, Standard deviation.

We investigated the association between the task frequency and rated importance, as well as an association with other variables such as level of education and number of years as a CRN. Differences between groups of experience in the CRN role were evaluated using analysis of variance – ANOVA test. To examine the relationship between the performed tasks and the number of years working as CRN, one‐way ANOVA was applied (Appendix S2). Games Howell post hoc test was used for pairwise comparison between the groups within each item. The level of statistical significance adopted was *p* < 0.05.

## 4 RESULTS

The majority of the participants were women representing various regions of Sweden. Their current work titles differed, such as RN, RN with specialist functions, specialist nurse, CRN and project or study coordinator. They worked full (47%) or part‐time (53%) as a CRN. Fewer than half (44%) stated that a job description was available. Only a fourth of the respondents stated that they had a professional development plan drawn up. Their education was mostly described as informal (*n* = 137), and the majority reported that they had taken the initiative to attend courses by themselves, such as ethics in research, biostatistics, data management and drug development courses. A minor part (*n* = 24) reported that they had formal education such as university‐led courses in clinical trials from 7.5 to 15 ECT (credits) (European Credit Transfer and Accumulation System, in higher education institutions. On average, one ECTS credit point equals between 25 and 30 working hours. (https://ec.europa.eu/assets/eac/education/ects/users‐guide/index_en.html, ECTS, 2015).

Many (*n* = 118), though, reported that they had attended one to five research courses the previous year; a few (*n* = 9) had attended six courses or more. Some CRNs (*n* = 34) reported that they had not attended any course during the last year. Many also highlighted the need for improved education, and if a specialist education to become a CRN existed, a majority (*n* = 124) would attend it.

**FIGURE 2:**
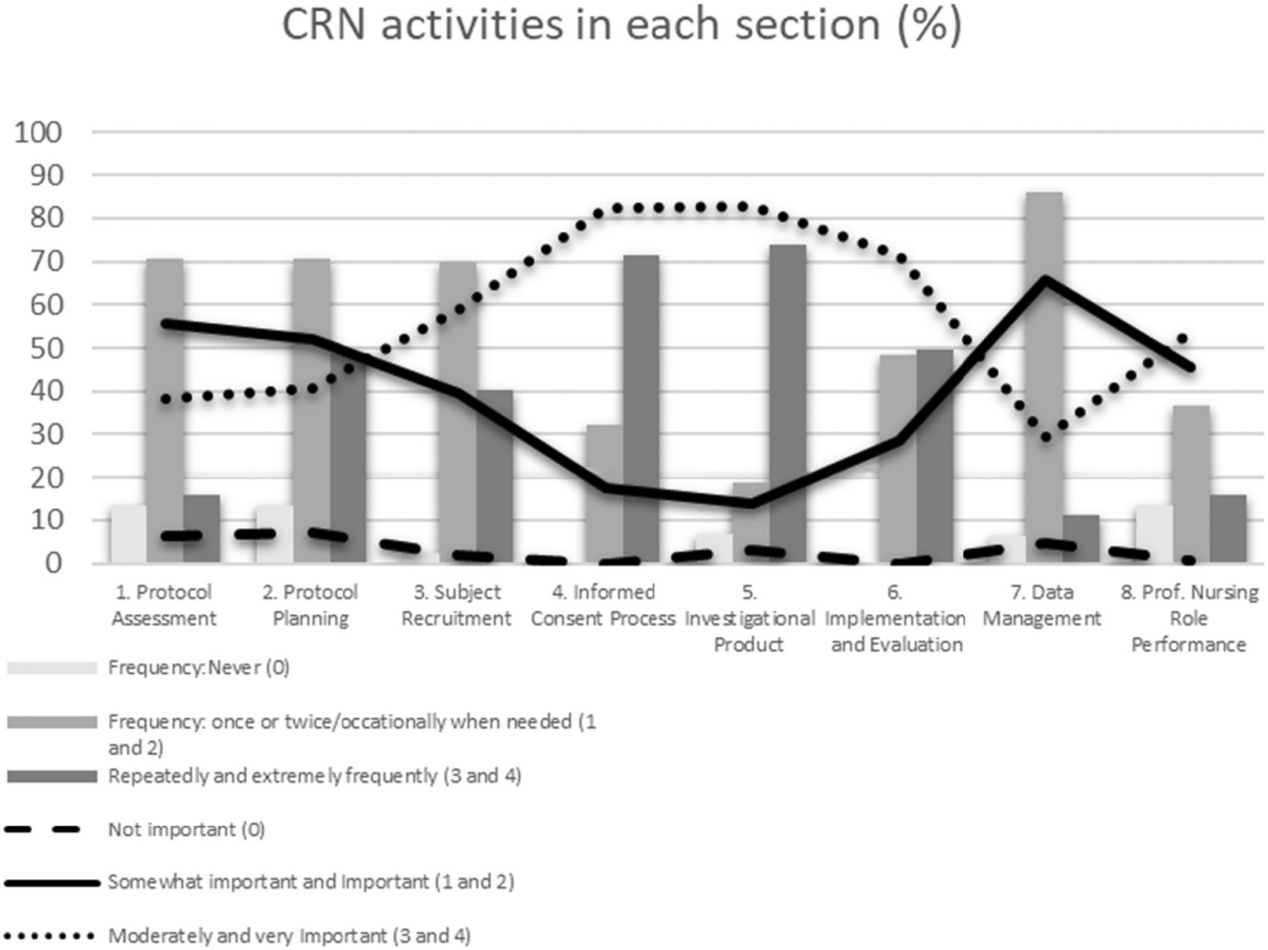
Frequency and importance of activities related to the role of the 161 Swedish CRNs

The CRNs reported that they were involved in pre‐study activities, study implementation and evaluation to various degrees. As shown in Figure 2, the most frequently performed activities concerned informed consent and managing the investigational products, which also were rated as most important by the CRNs. In general, the participants rated the importance of activities higher than the frequencies. Protocol planning and assessment were less frequently performed and rated as less important. The most and least frequently performed study tasks are presented in Table 3. The CRNs reported that they were often involved in practical work with inclusion, case report forms and study logs but seldom in budget issues, ethics applications and reporting of the results. In Table 4, the items assessed to be most and least important are listed.

### 4.1 Protocol assessment

The result showed that the activities concerning the assessment of protocol under development were performed less frequently in comparison to the activities in other sections. These activities are related to identifying concerns about the study design with the focus on the well‐being and security of the study patients. Assessing study budget and economics were seldom an activity for the CRNs and were also viewed as less important.

### 4.2 Protocol planning

Participating in study initiation meetings and determining that all required documents were in place before study start were the activities the CRNs performed most frequently. They were very seldom involved in preparing documents for the Ethics Review Committee.

### 4.3 Subject recruitment

In recruiting and screening potential study patients, the most frequent activities were related to maintaining screening logs and monitoring for correct inclusion. This was also graded with high importance. They seldom developed recruitment materials such as print materials and websites. Developing study‐related documents was something they also scored as of the lowest importance.

### 4.4 Informed consent process

Regarding the process of informed consent, securing written consent before participation in the study was the most frequent and important activity. Assessing the goals and purposes of the study patients in participating was rated with low frequency and low importance in their role as CRNs.

### 4.5 Investigational product

Regarding the investigational product, activities concerned with handling, ordering, accurately documenting and administering the study drugs, and maintaining accountability for the investigational product, were rated most frequently performed as well as most important.

### 4.6 Implementation and evaluation

Regarding activities related to implementing study‐related procedures and evaluating those, scheduling and performing procedures and tests according to protocol requirements were the most performed activity and rated with the highest importance among the CRNs. Performing psychosocial assessments of the subject/family was less frequently performed and graded lower in importance.

### 4.7 Data management

Among activities related to managing data in all forms, such as source data and records for collecting study data, and being a part of audits and inspections, the most frequently performed activity concerned entering data into case report forms and confirming its accuracy, that is, that data were input correctly, while preparing final written reports for the ethical review authority or for sponsors, was an activity they seldom performed.

### 4.8 Professional nursing role performance

This section related to how the CRNs performed activities based on their professional role, the most frequent activities concerning performance appraisals of their own practice and role performance in relation to peers or supervisor, identifying areas of strength and areas for further development. The lowest frequent activity concerned identifying clinical problems of research, something they performed only once or twice a year or occasionally.

### 4.9 Professional nursing role perception

In this section, the analysis showed that the CRNs strongly agreed or agreed (78.9%) that they felt competent and secure in their role. They also strongly agreed/agreed on having good communication with study patients and their families (97.5%) as well having good communication with healthcare personnel and the research team (94.4%). Approximately 98% strongly agreed or agreed that their work as a CRN was independent. In terms of acceptance of the role by colleagues, they reported high acceptance from the physicians (88.2%) but lower acceptance by other nurses not working as CRNs (55.9%).

### 4.10 Tasks related to the CRNs' work experience

Comparisons were made to identify differences between groups, that is, reported tasks and years of work experience as a CRN; see Appendix S1. Post hoc tests of sections and individual items (data not shown) showed that more experienced CRNs conducted some tasks more frequently than less experienced ones. Highly experienced CRNs (>15 years of experience) more often worked with all investigational product tasks, such as educating study patients, than did less experienced nurses (<10 years of experience). Regarding the frequency of implementation and evaluation and frequency of professional nursing role performance, post hoc tests could not find significant differences between the groups.

## 5 DISCUSSION

This study is the first scientific report on the tasks of CRNs in Sweden. The results show that a majority of the participating CRNs are involved in all procedures in the research process, not only the practical parts of clinical research studies. They contribute their knowledge in protocol planning and clinical study implementation and evaluation, while they simultaneously are focusing on the well‐being and safety of the research participants.

The consent process, subject recruitment, implementation and evaluation had the highest frequency scores. Similar results were shown by Nagel and Bonner (2010) through the Children's Oncology Group (COG) in the United States and Canada, but results from Catania et al. (2012) indicated that Italian CRNs seldom were involved in the consent process. We found that CRNs in Sweden reported a broader involvement in the entire study procedure than the Italian CRNs, who mostly were involved in the practical tasks in clinical study implementation (Catania et al., 2012). A study from the United States (Bevans et al., 2011) aiming to define the CRNs' role and to distinguish the role from the tasks of a clinical research coordinator showed results similar to those presented in this study. Additionally, in line with our results that the CRNs were seldom involved in study budget issues or in finding new areas for research as well as applications to ethics committee, a study from Ireland (Shilling & Hyland, 2019) also reported that ethics applications and study development were less common activities performed by CRNs. This can be explained by the application process to the Swedish ethical review boards, whereby the principal investigators are individually responsible for applying for approval when conducting clinical studies and are responsible for the study costs.

Furthermore, our results show that the respondents in general rated the importance of activities higher than the frequencies. This is similar to findings in studies from both Italy and Australia, indicating that the respondents are aware of their role as an important member of the research team (Catania et al., 2012; Wilkes et al., 2012).

Most of our respondents felt competent in their CRN role. They had long experience in both the RN and CRN roles, which probably contributed to their feeling of proficiency. However, they felt more support and acceptance from the physicians than from nursing colleagues. This result is similar to Spilsbury et al. (2008), who reported that British CRNs experienced that other nursing colleagues, RNs, were not familiar with the research process and regulations surrounding research and did not understand the CRNs' work. According to Höglund et al. (2010), Swedish CRNs described themselves as an unknown group of personnel at hospitals. Better knowledge and clarification of the role could facilitate communication with study personnel and other staff at clinics who are involved in clinical studies.

The education to become a CRN is mostly informal, and CRNs seldom have a job description or professional development plan. The majority of the respondents were positive towards attending a specialist education for CRNs if it had been available. Special programmes and education for CRNs have been developed or are moving towards development in countries such as the United Kingdom and Ireland and also in the United States, where CRNs have been recognized as a nursing speciality (Cline & Showalter, 2020; Hastings et al., 2012; Scott et al., 2013; Shilling & Hyland, 2019). As stated by Ness and Royce (2017), competence frameworks help to define the unique role a registered nurse has as a CRN, and, to establish a career pathway and to improve outcomes of clinical research studies. The result from this study is also important knowledge when conducting clinical research world‐wide where CRNs from different countries are involved. Furthermore, this knowledge about the Swedish CRN's role, makes it possible to adjust the research process in international multi centres' studies, and thereby, ensure the quality of the clinical research. Additionally, it provides new knowledge useful in further work to achieve an international consensus regarding the CRN's role and its definition.

According to Meleis' transition theory, a transition process occurs when a role is changing. Role change requires the incorporation of new knowledge and behaviours, which leads to a change in identity in social contexts/structures. How the role change is implemented depends on context, culture, individual factors, support and resources (e.g. knowledge and experience). One type of transition described by Meleis (2010) is the organizational transition that occurs as a result of organizational structures changing in policies and practices within the healthcare environment and affecting the lives of individuals who work in that environment. Such transitions may also affect patients, in both access and quality of care received (Meleis, 2010). The transition among nurses who become CRNs can create uncertainty, particularly when the role is unclear, leading to struggles with their professional identity (Tinkler et al., 2018). This study contributes to clarifying the activities that are performed in the role as a CRN. This knowledge could facilitate the adaptation to the new role and increase interest in becoming a CRN.

Most respondents in this study were nurses with long experience in nursing and as CRNs. The results indicate that the responsibilities and activities of CRNs differ with experience. Having a long experience in nursing may contribute to empowerment, thus making it easier to take on unfamiliar tasks and new responsibilities. This result is similar to findings by Shilling and Hyland (2019), who investigated the CRNs' role in Ireland. Summed up, the Swedish CRNs report that they are *active research players in the research team throughout the entire study process with a focus on the patient*'*s well‐being and safety*. This sentence, the essence of the study, could be used as a definition of the Swedish CRN.

## 6 METHODICAL CONSIDERATIONS

The study is limited by the lack of information on how large the entire CRN population is in Sweden. Whether the attempts to reach the whole study population of registered nurses working in the role of CRNs have been successful cannot therefore be verified. It is also not possible to assess the exact response rate, because we do not know how many of the non‐responders are CRNs. However, we have a broad range of respondents of different ages and with different experiences from different parts of Sweden, and therefore believe that the results are valid for Swedish CRNs in general. Different employment settings and varying titles are issues that make it hard to find the population of CRNs working in Sweden. The CTNQ‐SWE contains a large number of questions, taking a long time to answer, and respondents may thereby have been discouraged from answering the questionnaire. However, the number of participants (*n* = 161) is relatively high in comparison to other studies using versions of the same questionnaire, for example, in Italy of *n* = 30 (Catania et al., 2012) and in Australia *n* = 85 (Wilkes et al., 2012). A key strength of this study is the use of a validated and reliable instrument that has been translated and tested in the Swedish context, which supports the empirical findings.

## 7 IMPLICATIONS

This study contributes to clarifying the activities performed in the role of CRNs in Sweden. The findings have several practical implications for strengthening the quality of research in Sweden through, for example, education and support for registered nurses who want to work as CRNs. It could lead to development of education directed to CRNs, such as courses in the research process, ethics, data management, coordination and communication. The study could also be important to acknowledge the nursing profession, to develop competence frameworks and to create a potential career pathway for RNs. A suggested strategy for stakeholders to improve the quality and quantity of clinical research in Sweden is to strengthen and develop the role of CRNs.

## 8 CONCLUSION

Most CRNs in Sweden are involved in all procedures in the research process from protocol planning to clinical study implementation and evaluation while also focusing on the well‐being and safety of the research participants. The findings contribute to clarifying the CRN role in Sweden. The results could be used to prepare and make the transition easier for RNs who want to become CRNs. Knowledge about the educational needs as well as the role description can be used by the clinical research enterprise around the world, research nodes, healthcare organizations and other research actors aiming to support and develop the CRNs in their role.

## AUTHOR CONTRIBUTIONS

The authors confirm contribution to the paper as follows; Conception and Design: BL, NO. Acquisition of data: BL. Analysis and interpretation of data: BL, NO, JS, SH, ÅH. Drafting the manuscript: BL. Revising critically for important intellectual content: BL, NO, JS, SH, ÅH. All authors reviewed the results and approved the final manuscript.

All authors have agreed on the final version and meet at least one of the following criteria [recommended by the ICMJE (http://www.icmje.org/recommendations/)]:

- substantial contributions to conception and design, acquisition of data or analysis and interpretation of data;
- drafting the article or revising it critically for important intellectual content.

## CONFLICT OF INTEREST

The authors declare that there is no conflict of interest.

## ETHICAL APPROVAL

The study followed the ethical principles of the Declaration of Helsinki (1996) and was approved by the regional Ethics Review Authority (Dno: 2017/67–31). All participants were given written information about the study by email, attached with a personal link to the questionnaire. The study information stated that participation was voluntary and that the result would be presented in group level, with contact information to the researcher if they had any questions or felt a need for more details. Instead of signing an informed consent, it was explained at the front page of the online web‐questionnaire that they consented to participation by completing the questionnaire. No conflicts of interest have been identified.

## Supporting information


Appendix S1

Appendix S2

## Data Availability

Data available on request due to privacy/ethical restrictions. The data that support the findings of this study are not publicly available since the data consists of information that could compromise research participants’ privacy and consent.
