# Supplementary material for: Clarifying the role of clinical research nurses working in Sweden, using the Clinical Trial Nursing Questionnaire – Swedish version
Source: Nurs Open. 2022 Jun 2;9(5):2434–43. doi: 10.1002/nop2.1260 (PMC9374401; doi:10.1002/nop2.1260)
Supplement: Supplementary file 1 — Appendix S1 [file NOP2-9-2434-s001.doc]

STROBE Statement—Checklist of items that should be included in reports of ***cross-sectional studies***

|  | Item No | Recommendation |
| --- | --- | --- |
| **Title and abstract** | 1 | Indicate the study’s design with a commonly used term in the title or the abstract  *The title describes the study design as” Clarifying the role of clinical research nurses working in Sweden, using the Clinical Trial Nursing Questionnaire”* |
|  |  | (*b*) Provide in the abstract an informative and balanced summary of what was done and what was found  *The abstract describes aim/objectives, methods and findings, ( Page 1)* |
| Introduction | | |
| Background/rationale | 2 | Explain the scientific background and rationale for the investigation being reported  The introduction is described Page 2 row 5-23, background is described in (Page 2-3 paragraph 27-48) |
| Objectives | 3 | State specific objectives, including any prespecified hypotheses  *The specific aims of the study are stated in background page 3 paragraph44-48 methods and datacollection in the abstract* ***(****Page 1)* |
| Methods | | |
| Study design | 4 | Present key elements of study design early in the paper  *The studydesign are discussed in the methods -section in heading Design ,( Page 3. paragraph 57-59)* |
| Setting | 5 | Describe the setting, locations, and relevant dates, including periods of recruitment, exposure, follow-up, and data collection  *The setting is described in the procedure -section paragraphs 1 and 2 of the Methods section (Page 12) and in undersection, Datacollection who is described in section datacollection (page 4, paragraph 6-48)* |
| Participants | 6 | Give the eligibility criteria, and the sources and methods of selection of participants  Described in section datacollection Page 4 |
| Variables | 7 | Clearly define all outcomes, exposures, predictors, potential confounders, and effect modifiers. Give diagnostic criteria, if applicable  The outcomes of the study: in data analysis and result (Page 6 -12) |
| Data sources/ measurement | 8* | For each variable of interest, give sources of data and details of methods of assessment (measurement). Describe comparability of assessment methods if there is more than one group  *The source of data (the questionnaire) is described in metods section under subheading; Datacollection , (page -4 and 5)*  *The items and variables of the questionnaire were analyzed descriptively with mean values and standard deviations and methods such as T-tests One-way Anova used to calcualte differences and relationship between performed tasks and number of years working in the role as describe in methods section, subheading Data analysis*(Page 5- 6) |
| Bias | 9 | Describe any efforts to address potential sources of bias  Discussed under heading Ethical considerations (Page 6) and other sources can of course be due to sampling, but the reason for using snowball sampling in this unknown population has been addressed in methods section, subheading; data collection ( Page 12 ,paragraph 31- 50) and in Heading methodical considerations (P 12). |
| Study size | 10 | Explain how the study size was arrived at  It is addressed in detail in methods section, subheading; data collection (Page 3-5). |
| Quantitative variables | 11 | Explain how quantitative variables were handled in the analyses. If applicable, describe which groupings were chosen and why  In detail addressed in methods section, subheading; data collection and data analysis (Page 3-6) |
| Statistical methods | 12 | (*a*) Describe all statistical methods, including those used to control for confounding  In methods section, subheading; data analysis**.** (Page 5-6) |
|  |  | (*b*) Describe any methods used to examine subgroups and interactions  In datanalysis (Page 5-6) |
|  |  | (c*)* Explain how missing data were addressed  *No missing data occured* |
|  |  | (d) If applicable, describe analytical methods taking account of sampling strategy  *samplingsprocedure is described in section data collection (page 10****)*** |
|  |  | (*e*) Describe any sensitivity analyses  Described in data analysis (Page 5-6) |
| Results | | |
| Participants | 13* | (a)Report numbers of individuals at each stage of study—eg numbers potentially eligible, examined for eligibility, confirmed eligible, included in the study, completing follow-up, and analysed  *Both described in section datacollection* ***(3-5)*** *and in the first sentence in the Result section(****Page 6-11)*** |
|  |  | (b) Give reasons for non-participation at each stage  *N/A* |
|  |  | (c) Consider use of a flow diagram *figure 1.flow chart* |
| Descriptive data | 14* | (a)Give characteristics of study participants (eg demographic, clinical, social) and information on exposures and potential confounders  *Charecteristics about studypartcipants are given in results (****page 6-7****)* |
|  |  | (b) Indicate number of participants with missing data for each variable of interest  *The questionnaires design made it impossible to skip any question in order to complete it* |
| Outcome data | 15* | Report numbers of outcome events or summary measures  *Described in result section (PAGE 6-11)* |
| Main results | 16 | (*a*) Give unadjusted estimates and, if applicable, confounder-adjusted estimates and their precision (eg, 95% confidence interval). Make clear which confounders were adjusted for and why they were included  in the main result, the level of statistical significance are presented with p-value <0.05 |
|  |  | (*b*) Report category boundaries when continuous variables were categorized |
|  |  | (*c*) If relevant, consider translating estimates of relative risk into absolute risk for a meaningful time period  N/A |
| Other analyses | 17 | Report other analyses done—eg analyses of subgroups and interactions, and sensitivity analyses |
| Discussion | | |
| Key results | 18 | Summarise key results with reference to study objectives  *Described both in result (****page 6-11)*** *as well in Section conclusion (****Page 15)*** |
| Limitations | 19 | Discuss limitations of the study, taking into account sources of potential bias or imprecision. Discuss both direction and magnitude of any potential bias  *Bias and conflicts are discussed in section ethical conciderations (****Page 6) and in methodical considerations(page 14)*** |
| Interpretation | 20 | Give a cautious overall interpretation of results considering objectives, limitations, multiplicity of analyses, results from similar studies, and other relevant evidence  *Discussed in the section discussion result (****Page 12-14*** *)and in Heading Implication and Conclusion (Page 25)* |
| Generalisability | 21 | Discuss the generalisability (external validity) of the study results  *Validity is discussed in result discussion methodical conciderations (****page 14****)* |
| Other information | | |
| Funding | 22 | Give the source of funding and the role of the funders for the present study and, if applicable, for the original study on which the present article is based  *No funding* |

*Give information separately for exposed and unexposed groups.

**Note:** An Explanation and Elaboration article discusses each checklist item and gives methodological background and published examples of transparent reporting. The STROBE checklist is best used in conjunction with this article (freely available on the Web sites of PLoS Medicine at http://www.plosmedicine.org/, Annals of Internal Medicine at http://www.annals.org/, and Epidemiology at http://www.epidem.com/). Information on the STROBE Initiative is available at www.strobe-statement.org.
