# Supplementary material for: Clarifying the role of clinical research nurses working in Sweden, using the Clinical Trial Nursing Questionnaire – Swedish version
Source: Nurs Open. 2022 Jun 2;9(5):2434–43. doi: 10.1002/nop2.1260 (PMC9374401; doi:10.1002/nop2.1260)
Supplement: Supplementary file 2 — Appendix S2 [file NOP2-9-2434-s002.docx]

### Appendix 2. Analyse of variance One-Way ANOVA with number of years working as Clinical research nurse (CRN)

| **Sections** | **Groups**  **Years as CRN** | **Sum of Squares** | **df** | **Mean Square** | **F** | **Sig** |
| --- | --- | --- | --- | --- | --- | --- |
| Protocol assessment frequency | Between Groups | 8.754 | 5 | 1.751 | 2.096 | 0.069 |
|  | Within Groups | 129.493 | 155 | 0.835 |  |  |
| Protocol assessment importance | Between Groups | 6.821 | 5 | 1.364 | 1.84 | 0.108 |
|  | Within Groups | 114.902 | 155 | 0.741 |  |  |
| Protocol planning frequency | Between Groups | 6.893 | 5 | 1.379 | 1.494 | 0.195 |
|  | Within Groups | 143.029 | 155 | 0.923 |  |  |
| Protocol planning importance | Between Groups | 5.504 | 5 | 1.101 | 1.15 | 0.336 |
|  | Within Groups | 148.372 | 155 | 0.957 |  |  |
| Subject recruitment frequency | Between Groups | 1.883 | 5 | 0.377 | 0.643 | 0.667 |
|  | Within Groups | 90.817 | 155 | 0.586 |  |  |
| Subject recruitment importance | Between Groups | 3.507 | 5 | 0.701 | 1.237 | 0.295 |
|  | Within Groups | 87.898 | 155 | 0.567 |  |  |
| Informed consent process freq | Between Groups | 2.279 | 5 | 0.456 | 1.294 | 0.269 |
|  | Within Groups | 54.596 | 155 | 0.352 |  |  |
| Informed consent process imp | Between Groups | 1.462 | 5 | 0.292 | 0.901 | 0.482 |
|  | Within Groups | 50.265 | 155 | 0.324 |  |  |
| Investigational product frequency | Between Groups | 18.228 | 5 | 3.646 | 3.346 | 0.007 |
|  | Within Groups | 168.859 | 155 | 1.089 |  |  |
| Investigational product importance | Between Groups | 5.133 | 5 | 1.027 | 1.548 | 0.178 |
|  | Within Groups | 102.17 | 154 | 0.663 |  |  |
| Implementation evaluation freq | Between Groups | 6.563 | 5 | 1.313 | 2.696 | 0.023 |
|  | Within Groups | 75.459 | 155 | 0.487 |  |  |
| Implementation evaluation imp | Between Groups | 2.326 | 5 | 0.465 | 1.06 | 0.385 |
|  | Within Groups | 68.06 | 155 | 0.439 |  |  |
| Data management frequency | Between Groups | 3.468 | 5 | 0.694 | 1.601 | 0.163 |
|  | Within Groups | 67.155 | 155 | 0.433 |  |  |
| Data management importance | Between Groups | 3.305 | 5 | 0.661 | 1.009 | 0.415 |
|  | Within Groups | 101.572 | 155 | 0.655 |  |  |
| Professional nursing role performance freq | Between Groups | 9.17 | 5 | 1.834 | 3.454 | 0.005 |
|  | Within Groups | 82.291 | 155 | 0.531 |  |  |
| Professional nursing role performance imp | Between Groups | 3.707 | 5 | 0.741 | 1.042 | 0.395 |
|  | Within Groups | 110.236 | 155 | 0.711 |  |  |
